# Lycopene improves the efficiency of anti-PD-1 therapy via activating IFN signaling of lung cancer cells

**DOI:** 10.1186/s12935-019-0789-y

**Published:** 2019-03-21

**Authors:** Xiufeng Jiang, Hui Wu, Wei Zhao, Xiao Ding, Qian You, Feng Zhu, Meifang Qian, Ping Yu

**Affiliations:** Department of Respiratory Medicine, The Fifth People’s Hospital of Wuxi City, Wuxi, 214016 China

**Keywords:** Lung cancer, Lycopene, Programmed death-1 receptor (PD-1), Interferon (IFN) γ, Interferon-regulatory factor (IRF) proteins

## Abstract

**Background:**

Monoclonal antibodies targeting programmed death-1 receptor (PD-1) and its ligand (PD-L1) have been developed to treat cancers including lung cancer. In this study, we aimed to investigate whether lycopene could promote the effect of anti-PD-1 treatment on lung cancer.

**Methods:**

Tumor formation assay was conducted. Immune reactions were assessed by detecting several cytokine levels using enzyme-like immunosorbent assay. T cell activity was analyzed using cytometry. The mechanism of lycopene action was investigated using Western blot, quantitative real-time polymerase chain reaction and bisulfite sequencing analysis.

**Results:**

After the mice injected with Lewis lung carcinoma (LLC) cells were sacrificed, we found that combined lycopene and anti-PD-1 reduced the tumor volume and weight compared to control treatment. Cell apoptosis in the tumor tissues was significantly enhanced in mice with combined lycopene and anti-PD-1 treatment in comparison with those of either lycopene or anti-PD-1 alone. Furthermore, lycopene could assist anti-PD-1 to elevate the levels of interleukin (IL)-1 and interferon (IFN) γ while reduce the levels of IL-4 and IL-10 in the spleen of mice injected with LLC cells. Lycopene treatment increased the CD4+/CD8+ ratio in the spleen and promoted IFNγ-expressing CD8+ T cells in tumor tissues. Upon IFNγ stimulation, lycopene diminished PD-L1 expression via activating JAK and repressing phosphorylation of AKT.

**Conclusion:**

Our results have demonstrated that lycopene could be used as a potential adjuvant drug to synergistically improve the efficiency of anti-PD-1 therapy.

**Electronic supplementary material:**

The online version of this article (10.1186/s12935-019-0789-y) contains supplementary material, which is available to authorized users.

## Background

Lung cancer is one of the most malignant tumors and the leading cause of cancer death worldwide [1, 2]. Only 18% of patients survive from lung cancer [2]. However, most patients are diagnosed in advanced stages with less than 5% patients survive for 5 years [3]. The first-line treatment depends on genetic aberration of patients. For example, drugs targeting epidermal growth factor receptor (EGFR) and translocation of anaplastic lymphoma kinase (ALK) have been applied in patients with EGFR mutation or ALK translocation [4, 5]. However, for patients without those oncogenic drivers, cytotoxic chemotherapy is commonly applied. Nevertheless, poor prognosis and drug resistance are still obstructions for the efficiency of current treatments. Therefore, recent attempts have begun to focus on treating lung cancer by modulating the immune reactions.

Recently developed drugs that regulate specific immune checkpoints, and monoclonal antibodies targeting programmed death-1 receptor (PD-1) and its ligand (PD-L1), have all showed impressive anti-tumor effects [6–9]. PD-1 is a cell surface protein containing 288 amino acid. It has two ligands, PD-L1 and PD-L2. Studies have evidenced that high expression of PD-L1 has been observed in numerous cancers and is associated with poor outcomes [10]. PD-L1 expression in tumor cells facilitates their escape from immune system surveillance. Among all patients with advanced non-small cell lung cancer (NSCLC), more than 20% showed PD-L1 expression in at least half of tumor cells [8, 11]. Therefore, improving the efficiency of anti-PD-1 therapy could yield promising outcomes.

Previous results have indicated that lycopene exerts anti-tumor effects by altering the methylation of genes through inhibiting DNA methyltransferase (DNMT) enzyme activity [12, 13]. DNMT inhibitors have been shown to enhance the efficacy of anti-PD-1 therapy in the treatment of lung cancer by activating interferon (IFN) signaling [14]. Here, we sought to investigate whether lycopene could promote the effects of anti-PD-1 treatment on lung cancer. A series of results confirmed that lycopene promoted anti-PD-1 therapeutic efficiency of lung cancer by promoting IFNγ-expressing CD8+ cells infiltrated in tumor tissues and increasing IFNγ expression in tumor cells.

## Methods

### Tumor formation assay

The effect of combined therapy was detected in vivo. Lewis lung carcinoma (LLC) cells (1 × 10^6^) were injected in the rear flak of C57BL/6 mice. Anti-mouse PD-1 antibodies (6 mg/kg) were administrated by intraperitoneal injection 3 days apart for 4 times. Lycopene (40 mg/kg) was intraperitoneally injected daily. Tumor volume and weight were measured. Tumor volume (mm^3^) was calculated using (length × width^2^)/2. The animal study was approved by the Ethical Committee of the Fifth People’s Hospital of Wuxi City. Mice were sacrificed to obtain tumor tissues.

### Enzyme-like immunosorbent assay (ELISA)

The cytokine levels in the spleen were measured using ELISA. Briefly, the spleen of mice was obtained and minced in phosphate buffered saline. The levels of interleukin (IL)-2, interferon (IFN) γ, IL-4 and IL-10 were detected in triplicates by ELISA kits (Groundwork Biotechnology Diagnosticate, USA) according to the manufacturers’ instructions. The color absorbance was measured at 450 nm with an ELISA plate reader (SoftMax Pro 5.3).

### Flow cytometry assay

The spleen or tumor tissues were mechanically disrupted using a gentleMACS™ dissociator (Miltenyi Biotec, Bergisch Gladbach, Germany) in 4-(2-hydroxyethyl)-1-piperazineethanesulfonic acid buffer to isolate cells for flow cytometry. Cells were then stained with fluorescently-labeled antibodies including anti-CD8 PE (Biolegend, San Diego, CA, USA), anti-CD4 (L3T4) FITC (Biolegend, San Diego, CA, USA) and anti-IFN-γ (BD Biosciences, Franklin Lakes, NJ, USA). For apoptosis analysis, perforin and granzyme B were employed.

### Quantitative real-time polymerase chain reaction (qRT-PCR)

Total RNA was extracted from tumor tissues or LLC cells using Trizol reagent (TaKaRa, Dalian, China). For cDNA synthesis, 2 µg RNA was used and ThermoScript RT-PCR System (Invitrogen, Waltham, MA USA) was applied according to manufactures’ instructions. The expression levels of IFNβ, IFNγ, CXCL9, CXCL10, PD-L1, IRF1/7 and IRF3/8 were detected and normalized to GAPDH. The primers were listed in Table 1.

**Table 1:** Primer sequences used for real-time PCR

Gene	Forward primer (5′–3′)	Reverse primer (5′–3′)
*IFNβ*	AGCTCCAAGAAAGGACGAACA	GCCCTGTAGGTGAGGTTGAT
*IFNγ*	ACACTGCATCTTGGCTTTGC	GCTTTCAATGACTGTGCCGT
*IRF1*	GGCCGATACAAAGCAGGAGAA	GGAGTTCATGGCACAACGGA
*IRF7*	TCCAGTTGATCCGCATAAGGT	CTTCCCTATTTTCCGTGGCTG
*IRF3*	TACACTGAGGACTTGCTGGAGGT	AAGATGGTGGTCTCCTGATCC
*IRF8*	CGAGGTTACGCTGTGCTTTG	TTATGCTTGGCTCTGTGGGG
*PDL*-*1*	GCTCCAAAGGACTTGTACGTG	TGATCTGAAGGGCAGCATTTC
*CXCL9*	GGAGTTCGAGGAACCCTAGTG	GGGATTTGTAGTGGATCGTGC
*CXCL10*	CCAAGTGCTGCCGTCATTTTC	GGCTCGCAGGGATGATTTCAA
*Gapdh*	AGGTCGGTGTGAACGGATTTG	GGGGTCGTTGATGGCAACA

### Bisulfite sequencing analysis

The DNA methylation of IRF1/7 promoter was analyzed by bisulfite sequencing. Briefly, the genomic DNAs obtained from tumor tissues or cells were subjected to bisulfite treatment. The methylation status of IRF1/7 was determined by methylation specific PCR using AmpliTaq Gold (Perkin-Elmer, MA, USA). Methylation specific primers are listed in Table 2.

**Table 2:** Primers for methylation-specific PCR

Gene	Forward primer (5′–3′)	Reverse primer (5′–3′)
*IRF1 methylated*	TGAAAAGATGGTTTTAGGAGTTAGTC	GACTACGTACCGTCATTTCGAA
*IRF1 unmethylated*	AAAGATGGTTTTAGGAGTTAGTTGG	ACCAACTACATACCATCATTTCAAA
*IRF7 methylated*	TAGATTATTTGAGGTCGGGAGTTC	AAAAAAACAACTACACCGTTTACGT
*IRF7 unmethylated*	TAGATTATTTGAGGTTGGGAGTTTG	AAAAAACAACTACACCATTTACATT

### Western blot

Total proteins were extracted by cell lysis buffer (20 mM NaCl, 10% glycerol, 1% Triton X-100). To separate proteins, 10% sodium dodecyl sulfate polyacrylamide gel electrophoresis was employed. Proteins were then transferred to Immobilon-P membranes (Millipore, Bedford, MA, USA) and incubated with primary antibodies overnight at 4 °C. Anti-DNMT1, anti-DNMT-3a, anti-DNMT3b, anti-PD-L1, anti-pJAK2, anti-JAK2, anti-p-STAT3, anti-STAT3, anti-p-AKT, anti-AKT and anti-β-actin were purchased from Sigma (St. Louis, MO, USA). All primary antibodies were diluted at 1:1000. After incubating with secondary antibody, the membranes were developed with Enhanced Chemiluminescence Plus Western Detection kit (Amersham Pharmacia Biotech, Piscataway, NJ, USA). For all quantization results of Western blotting, densitometry analysis was used. The values were obtained by dividing the optical density values of interested proteins by it of β-actin. The results were counted and plotted.

### Statistical analysis

All data were presented as mean ± standard deviation (SD). Statistical analysis was performed using SPSS 19.0 (IBM., Chicago, IL, US) and Graphpad Prism 7 (Graphpad Inc., La Jolla, CA, US). Statistical significance was assessed using analysis of variance (ANOVA), unpaired t-tests or Mann–Whitney U-tests. p value of less than 0.05 was regarded as statistically significant.

## Results

### Lycopene improves the efficiency of anti-PD-1 therapy in NSCLC

**Fig. 1:**
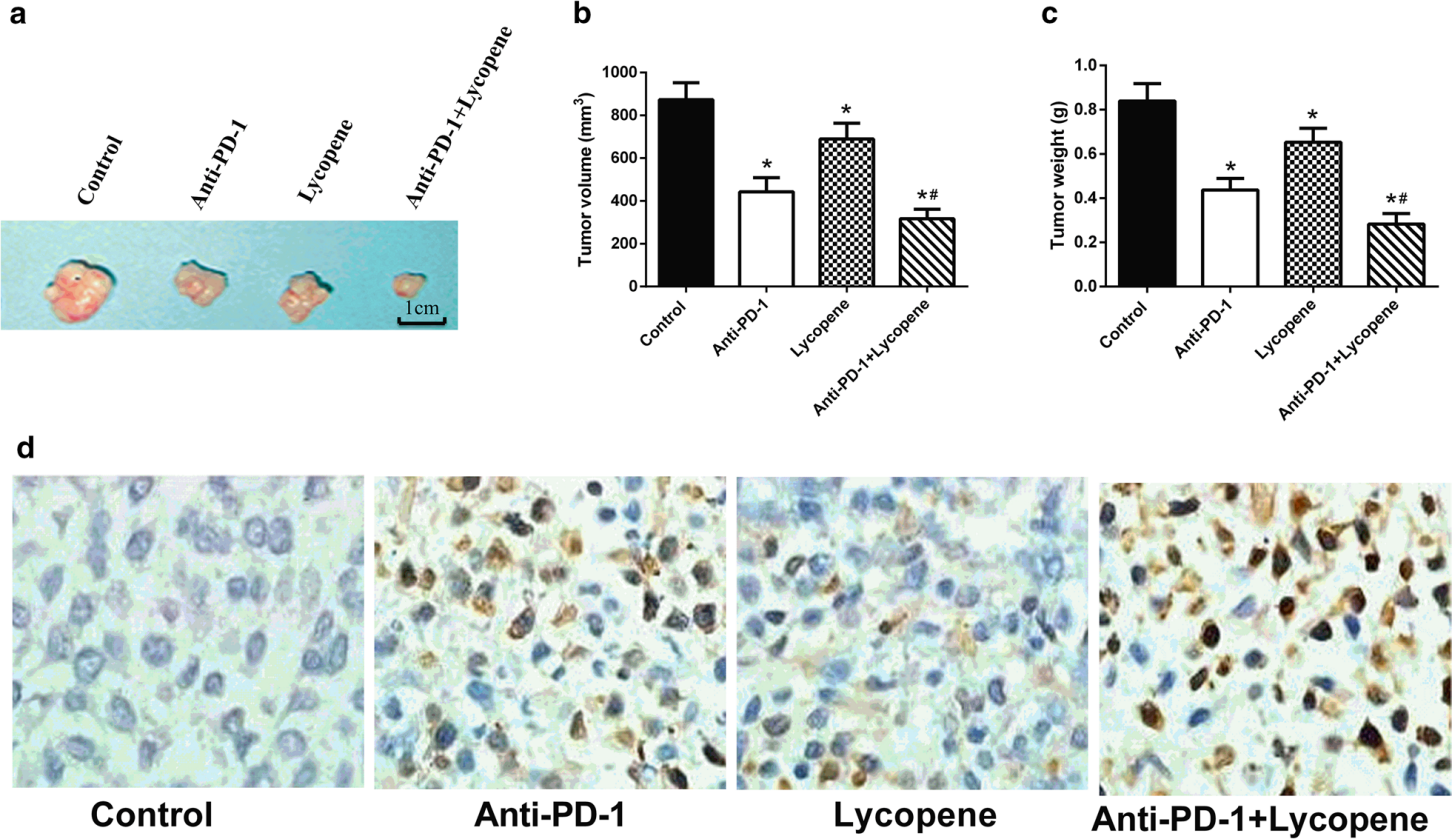
Lycopene improves the efficiency of anti-PD-1 therapy. 1 × 10^6^ LLC cells were injected in the rear flank of C57BL/6 mice. Lycopene (40 mg/kg) was intraperitoneally injected daily. Anti-mouse PD-1 antibodies (6 mg/kg) were administrated by intraperitoneal injection 3 days apart for four times. The mean tumor volume (mm^3^) and tumor weight (g) were measured (**a**–**c**). Tunnel staining (100 × 10) of apoptotic bodies of gastric tumors (**d**). *p < 0.05 vs. control, ^#^*p* < 0.05 vs. Anti-PD-1 (n = 6)

To investigate whether lycopene could enhance the efficiency of anti-PD-1 therapy, we performed tumor formation assay in the rear flank of C57BL/6 mice using LLC cells. Anti-PD-1 and lycopene treatments both inhibited tumorigenesis of LLC. Anti-PD-1 antibodies and lycopene were administrated by intraperitoneal injection. Surprisingly, when anti-PD-1 was combined with lycopene, tumor formation was further repressed (Fig. 1a, Additional file 1: Figure S1). The volume and weight of all tumors were also measured (Fig. 1b, c). Lycopene or anti-PD-1 treatment alone could reduce tumor volume and weight compared to untreated mice. When anti-PD-1 combined with lycopene was applied to mice, it showed more pronounced inhibitory effect on tumor growth. Additionally, cell apoptosis was measured in these tumor tissues. Although lycopene alone showed little effect on cell apoptosis, we found that lycopene combined with anti-PD-1 treatment significantly enhanced cell apoptosis compared to either lycopene or anti-PD-1 treatment alone (Fig. 1d).

### The combined lycopene and anti-PD-1 therapy alters the immune responses of mice and promoted death of tumor cells

**Fig. 2:**
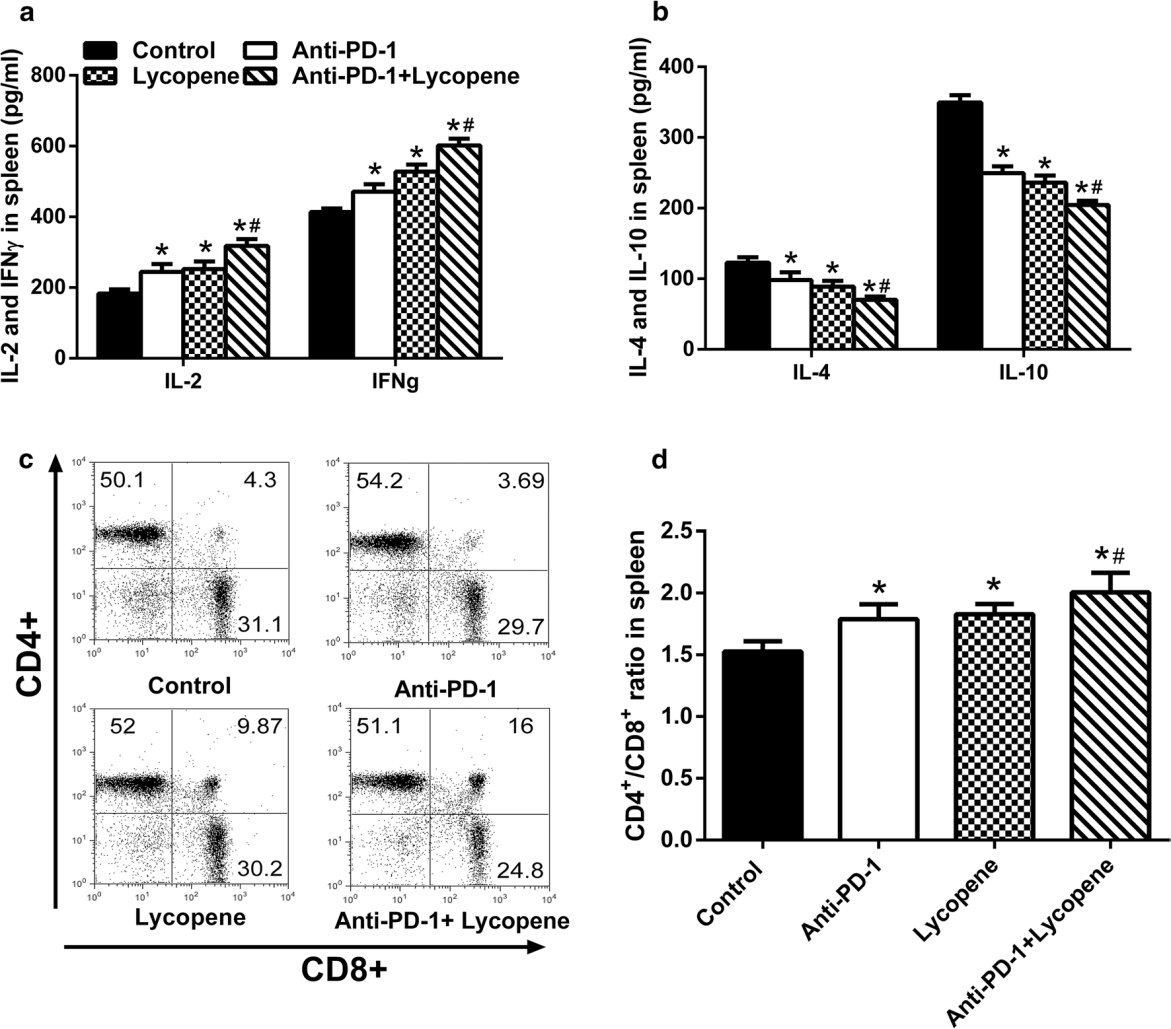
The combined therapy of lycopene and anti-PD-1 synergistically regulates the immune system. The inflammatory factors in the spleen were detected by ELISA (**a**, **b**). The CD4+/CD8+ ratio in the spleen was measured by flow cytometry analysis (**c**, **d**). IFNγ+/CD8+T-cells infiltrated in the tumor tissues were determined by flow cytometry analysis (**d**). *p < 0.05 vs. control, ^#^*p* < 0.05 vs. Anti-PD-1 (n = 6)

**Fig. 3:**
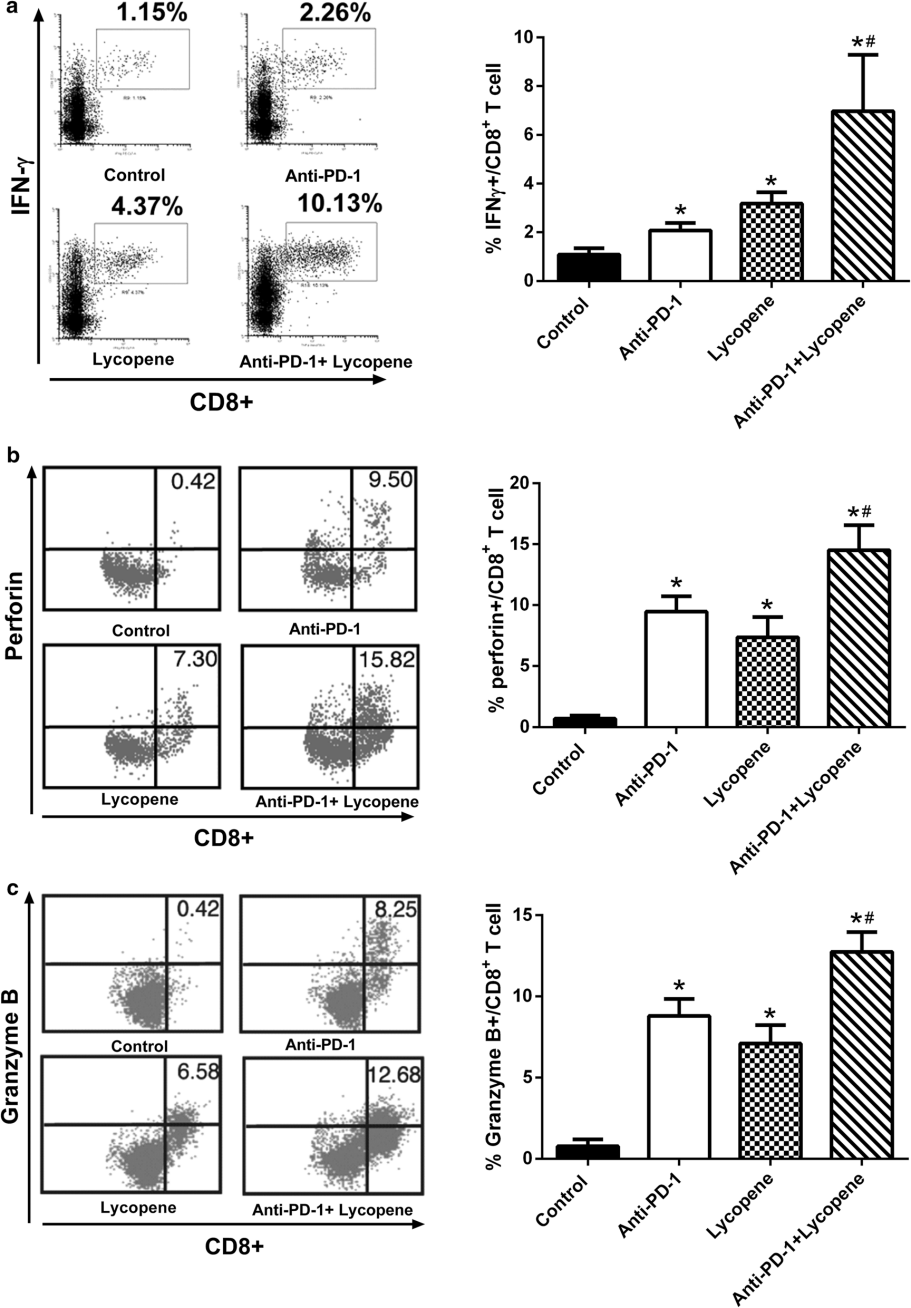
The combined therapy of lycopene and anti-PD-1 synergistically regulates cancer cell death cytokine. IFNγ+/CD8+ (**a**), perforin+/CD8+ (**b**), granzyme B+/CD8+ (**d**) population in tumor-infiltrated T cells was measured by flow cytometry analysis. *p < 0.05 vs. control, ^#^*p* < 0.05 vs. Anti-PD-1 (n = 6)

Since anti-PD-1 therapy prevents the escape of tumor cells from T cells, we then investigated the mechanism underlying how lycopene synergistically functioned with anti-PD-1. We tested the levels of IL-2, IFNγ, IL-4 and IL10 in the spleen and found that lycopene showed similar functions as anti-PD-1 in promoting the productions of IL-2 and IFNγ (Fig. 2a, b). These effects of lycopene and anti-PD-1 could be reinforced when combined together. In addition, it was observed that either anti-PD-1 or lycopene led to increased CD4+/CD8+ ratio in the spleen (Fig. 2c, d). Furthermore, we found that lycopene could synergistically function with anti-PD-1 to improve IFNγ expression of CD8+ cells infiltrated in the tumor tissues (Fig. 3a). In addition, we tested cell death in those tumor tissues by analyzing Granzyme B+/CD8+ and perforin+/CD8+ population using flow cytometry. It was found that combined use of lycopene and anti-PD-1 led to significant increase of cell death compared to either lycopene or anti-PD-1 treatment alone (Fig. 3b, c).

### Lycopene reduces the methylation levels of IRF1 and IRF7 promoters to promote IFNγ expression

**Fig. 4:**
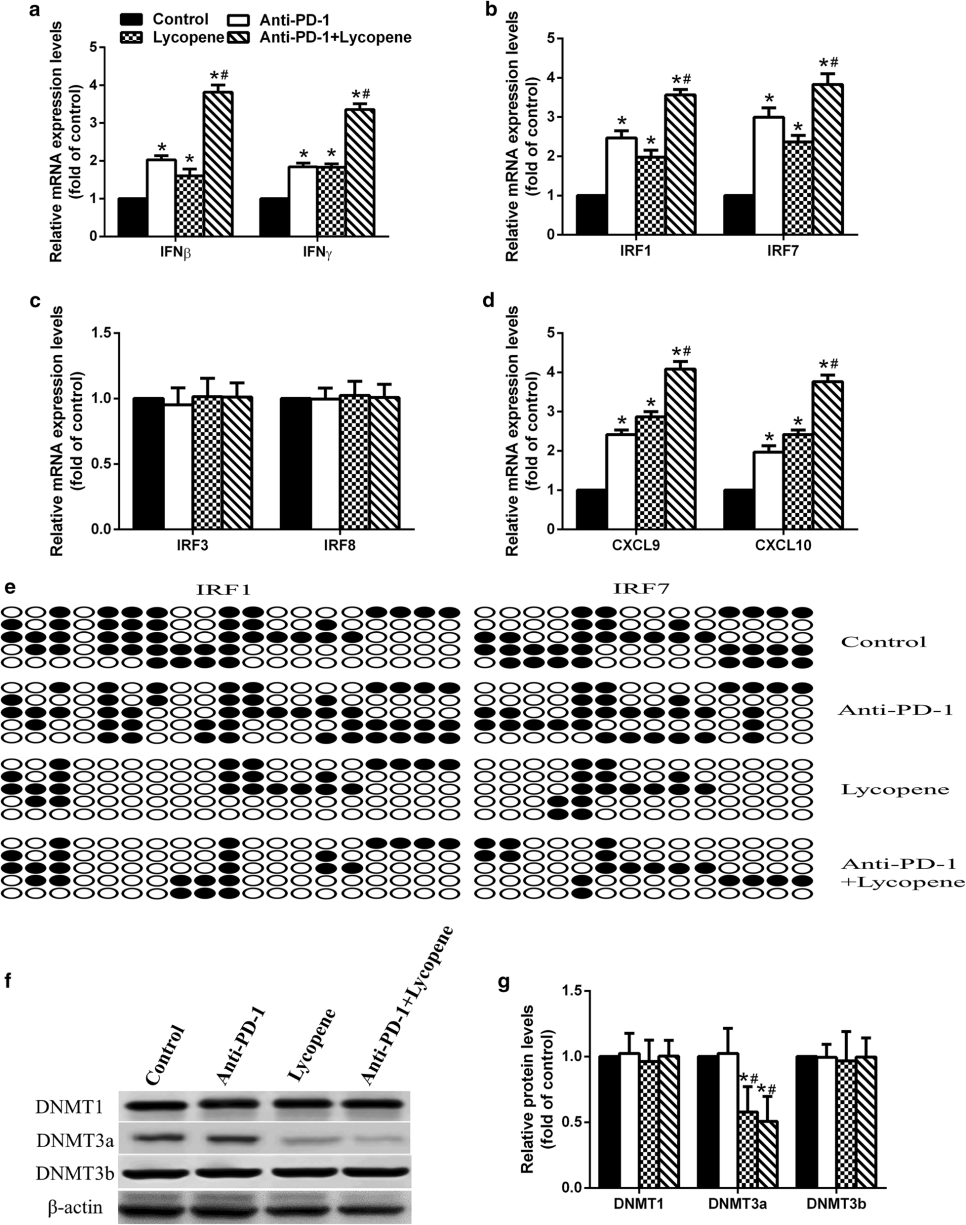
Lycopene reduces the methylation levels of IRF1 and IRF7 promoters to promote IFNγ expression. The IFN-related gene expressions were measured by qRT-PCR (**a**–**d**). After the treatment of lycopene and/or PD-1 therapy, the methylation status of IRF1/7 promoter regions were assessed by bisulfite sequencing analysis, (black circle, methylated cytosine of CpG; white circle, unmethylated cytosine of CpG) (**e**). The expression levels of DNMT1, DNMT3A, and DNMT3B in the tumor tissues were analyzed by Western blot (**f**, **g**). *p < 0.05 vs. control, ^#^*p* < 0.05 vs. Anti-PD-1 (n = 6)

We further investigated whether lycopene regulated the mRNA level of IFNγ. We found that, in the tumor tissue obtained from mice, lycopene significantly upregulated the mRNA levels of IFNβ, IFNγ, IRF1, IRF7, CXCL9 and CXCL10, whereas no effect was observed on IRF3 and IRF8 (Fig. 4a–d). To explore the mechanism underlying lycopene regulation on the expression of these factors, we further tested the promoter methylation levels of IRF1 and IRF7. It was observed that the methylation levels on the promoters of IRF1 and IRF7 showed decreased trend in the mice administrated with lycopene and anti-PD-1, compared to those in the untreated mice as well as mice with either lycopene or anti-PD-1 alone (Fig. 4e). We next measured the levels of DNMT1, DNMT3A and DNMT3B in these tumor tissues. It was found that the DNMT3a level was dramatically repressed when mice were treated with combined lycopene and anti-PD-1, compared to that of mice with lycopene or anti-PD-1 alone treatment (Fig. 4f, g). Interestingly, the methylation status of IRF3/8 promoter regions was not changed by the treatment of lycopene and/or PD-1 therapy (Additional file 1: Figure S2).

### The effect of lycopene on the expression of IFNγ-related genes in LLC cells

**Fig. 5:**
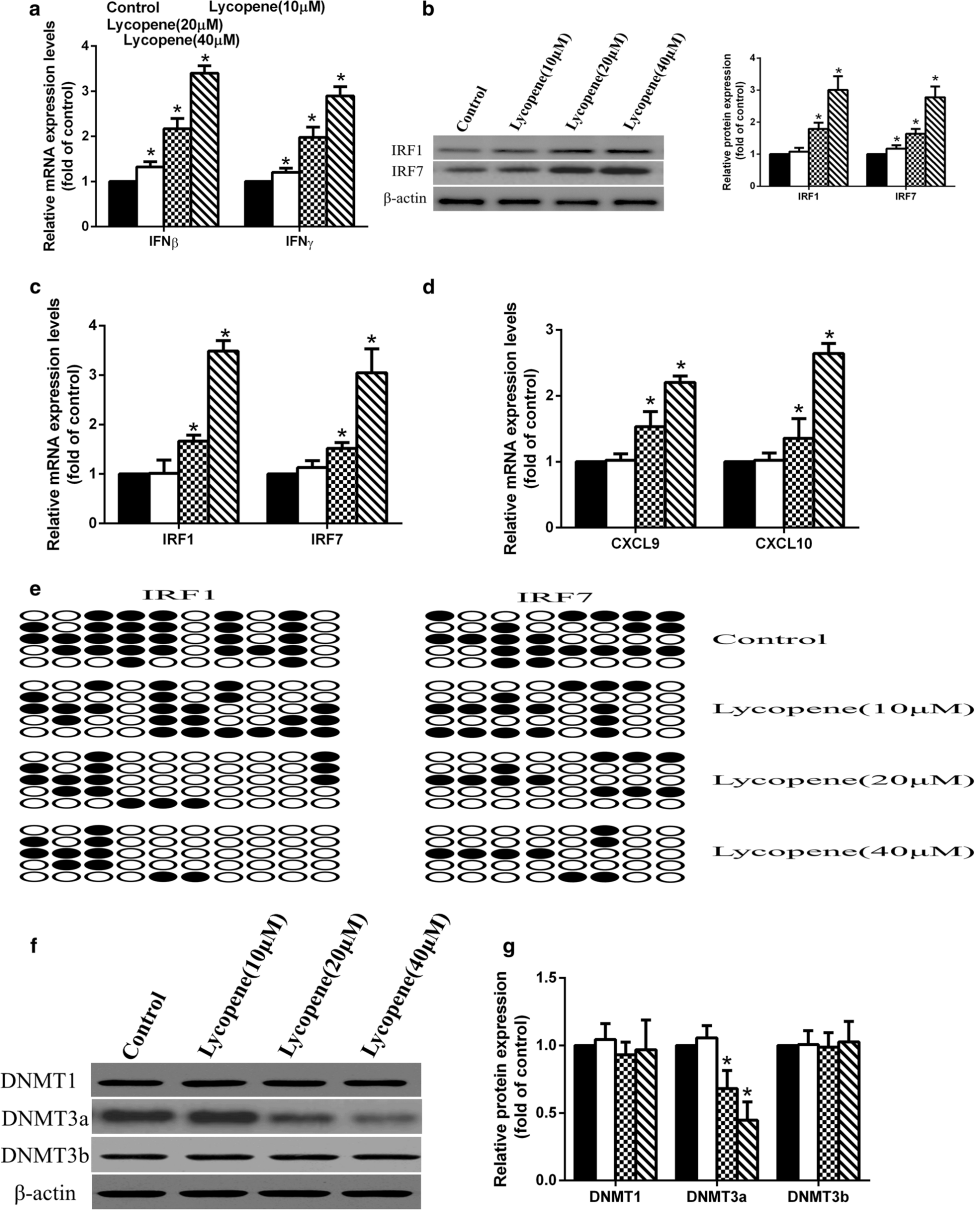
The effect of lycopene on the expression of IFNγ related genes in LLC cells. The IFN-related gene expressions were measured by qRT-PCR, and the protein expression of IRF1, IRF7 were measured by Western blot (**a**–**d**). After the treatment of lycopene, the change of methylation status of IRF1/7 promoter regions were assessed by bisulfite sequencing analysis, (black circle, methylated cytosine of CpG; white circle, unmethylated cytosine of CpG) (**e**). The expressions of DNMT1, DNMT3A, and DNMT3B in LLC cells were analyzed by Western blot (**f**, **g**). *p < 0.05 vs. control

We then tested the expression of IFNγ-related genes in LLC cells treated with or without lycopene. We found that lycopene remarkably increased the mRNA and protein levels of IRF1 and IRF7 in a dose-dependent manner (Fig. 5a, b). The mRNA levels of IFNβ, IFNγ, CXCL9 and CXCL10 were also upregulated by lycopene (Fig. 5c, d). Consistently, lycopene treatment repressed promoter methylation of IRF1 and IRF7 in a dose-dependent manner (Fig. 5e). The inhibitory effect of lycopene on DNMT3a was also observed in LLC cells (Fig. 5f, g).

### The effect and mechanism of lycopene on the expression of PDL-1 downstream of IFNγ signaling

**Fig. 6:**
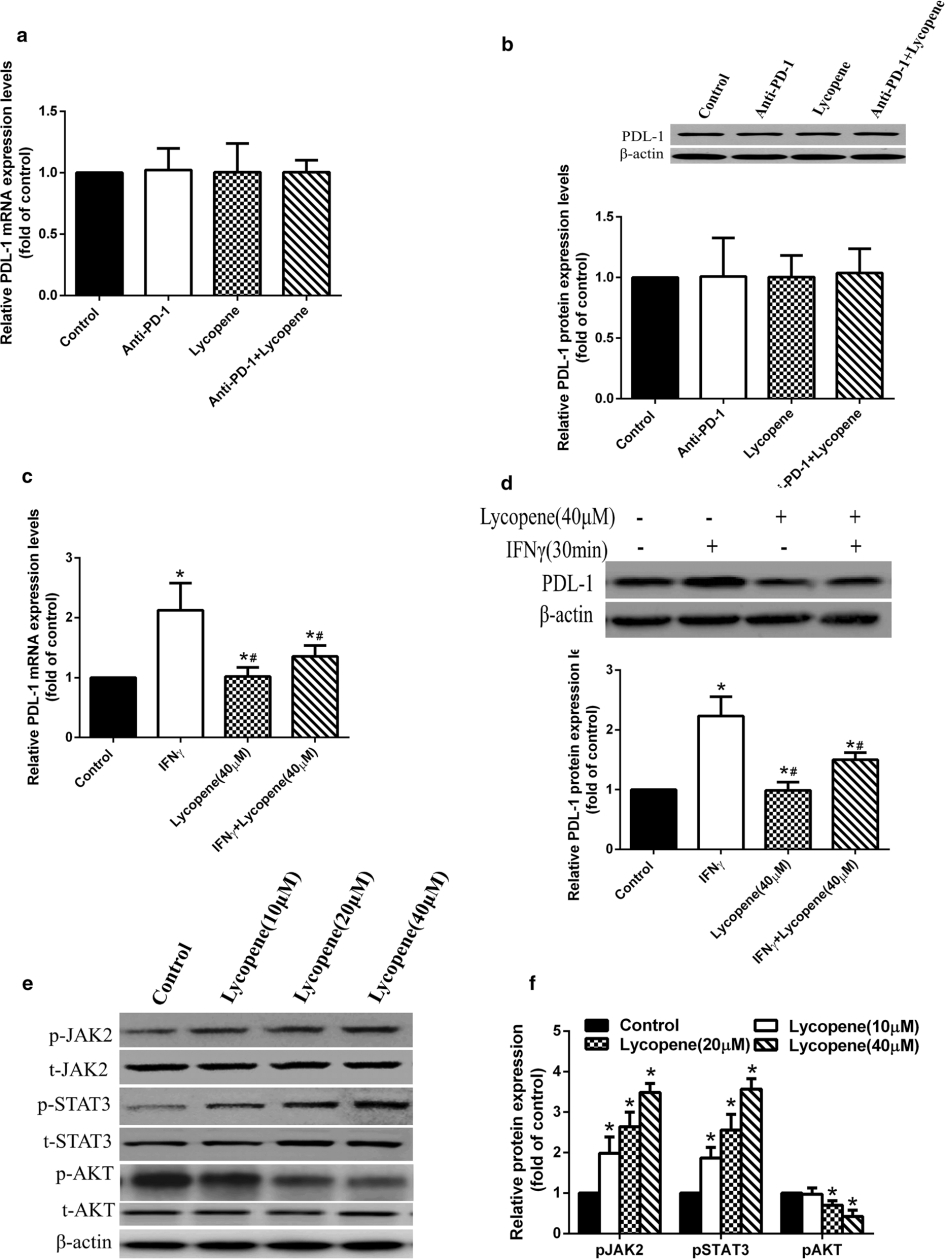
The effect and mechanism of lycopene on the expression of PDL-1 downstream the IFNγ signaling. The mRNA and protein expression of PDL-1 in tumor were detected by qRt-PCR and Western blot (**a**, **b**). Lycopene inhibited the increased expression of PD-L1 induced by IFNγ in LLC cells (**c**, **d**), *p < 0.05 vs. control, ^#^*p* < 0.05 vs. IFNγ (n = 6). Lycopene activated the JAK2 signal and inhibited AKT signaling to repress expression of PD-L1 in LLC cells (**e**, **f**), *p < 0.05 vs. control (n = 6)

IFNγ exerts anti-tumor function mainly through JAK2 signaling. It is also able to promote PDL-1 expression via AKT signaling. Therefore, we further tested the effect of lycopene on PDL-1 expression. No change was observed in the mice with or without lycopene treatment (Fig. 6a, b). Interestingly, when we stimulated LLC cells with IFNγ for 30 min, lycopene treatment showed inhibitory effects on PD-L1 expression (Fig. 6c, d). Additionally, we found that lycopene promoted JAK2 signaling but blocked AKT signaling (Fig. 6e, f). These conclusions were confirmed when using a PI3 K-AKT inhibitor, LY294002, which significantly repressed PD-L1 protein level in IFNγ and lycopene treated cells (Additional file 1: Figure S3).

## Discussion

About 20% of patients with advanced NSCLC showed responses to an antibody targeting PD-1, pembrolizumab [6, 15]. Investigations have demonstrated several factors that impact the sensitivity to pembrolizumab, including EGFR mutations, ALK rearrangements and mutational landscape [16]. Additionally, few patients show strong reactions to anti-PD-1. Therefore, it is urgent to improve the efficiency of anti-PD-1 therapy.

In the present study, we demonstrated that both anti-PD-1 and lycopene alone had the ability to repress tumorigenesis. When combining anti-PD-1 therapy with lycopene, their anti-tumor effect was significantly increased. Although there were a series of studies demonstrating that lycopene intake was associated with decreased risks of prostate, gastric, breast, and lung cancers [17–21], whether it could synergistically function with anti-PD-1 therapy has not been reported. Here, we found that lycopene synergistically exerts anti-tumor function with anti-PD-1. In addition, cell apoptosis and cell death were promoted in the tumors of mice treated with combined lycopene and anti-PD-1. These results suggest that lycopene promotes anti-PD-1 effects on cytotoxic CD8 cells, which on the other hand increases cell death.

Since lycopene has been reported to play a role in the immune system, it is possible that the increased efficiency of anti-PD-1 therapy by lycopene is mediated through its functions in regulating immune response. Previous evidence indicated that lycopene inhibited inflammatory responses through blocking IκB kinase, reducing Toll-like receptor 4 expressions and the productions of several NF-κB-related cytokines such as Tumor necrosis factor-α, IL-1β and IL-6β [21, 22]. It is known that blocking PD-1 unleashes the T cells to target tumor cells. Therefore, based on the surprising effects of lycopene on anti-PD-1 treated lung cancer, we further investigated whether lycopene affected T cell activities. As expected, the productions of IL-2 and IFNγ were significantly increased in the spleen of mice treated with lycopene, whereas IL-4 and IL-10 were inhibited. The ratio of CD4+/CD8+ cells in the spleen was enhanced in lycopene-treated mice compared to untreated mice, indicating that lycopene is able to promote immune response by enhancing the activation, growth and differentiation of T cells, as well as impact T helper (Th)1/Th2 drift. In addition, increased IFNγ-expressing CD8+ cells in tumor tissues suggests that lycopene may disrupt the functions of PD-1 and PD-L1 to reduce tumor escaping from the immune system.

Interestingly, we found that lycopene promoted the mRNA expression levels of IFNγ and IFNβ and their downstream gene expression including IRF1, IRF7, CXCL9 and CXCL10. It is known that IRF1 is an important factor involved in apoptosis, which can be stimulated upon stimuli such as IFNγ and activated IFNβ gene [23]. Loss of IRF1 expression or function has been reported to be associated with several human cancers such as breast cancer and gastric cancer [24–26]. Another IRF family protein, IRF7, also plays critical roles in tumors. A previous study reported that silencing IRF7 could assist tumor cell to escape from the immune system, leading to increased tumor metastasis [27]. Essentially, IRF7 is also well-known by its regulatory role in the induction of IFNα/β genes [28]. Therefore, the elevated expression of IFNβ may be caused by the impact of lycopene on IRF7. Although IRF3 is also a master regulator for Type I IFN expression, we did not observe a significant change of IRF3 expression upon lycopene treatment. Additionally, no difference in IRF8 expression was exhibited upon lycopene treatment. It is known that IFNγ induces IRF8 expression [29], and IRF8 expression could be repressed by DNA methylation [30]. Here, we found that lycopene treatment diminished the methylation levels of IRF1/7 promoters but not IRF8. It may indicate that lycopene may not directly function in IRF1/7 promoter methylation. However, we observed that lycopene significantly repressed the expression level of DNMT3a but did not affect DNMT1 and DNMT3b. Previous studies have demonstrated that DNMT3a is a negative regulator of IRF8 [31]. Therefore, this result further indicates that lycopene treatment affecting the mRNA levels of IRF1 and IRF7 may only be caused by its role in IFNγ. Further study is needed to explain how lycopene regulates DNMT3a but showed no effect on IRF8.

The role of IFNγ in anti-tumor immunity could be highlighted by resistance of tumors to interferons that leads to their evasion from the immune system [32]. Recently, immune checkpoint blockade (ICB) has been implicated in cancer treatments, which blocks inhibitory receptors on the surface of intra-tumoral effector T cells [33, 34]. Results that ICB could induce production of intra-tumoral IFNγ in mouse models and human patients, as well as the dependence of tumor infiltration on IFNγ receptor by immune cells [35, 36], indicated a key role of IFNγ in rejecting tumor. Studies from patients resistant to anti-PD1 therapy have also provided strong support for ICB-induced IFNγ action directly on tumor cells [37, 38]. It would be interesting to further test these possibilities in the context of NSCLC.

T cell-secreted IFNγ was reported to mediate the induction of PD-L1 expression in melanoma microenvironment [39]. In this study, we found that lycopene was able to decrease both mRNA and protein levels of PD-L1 upon IFNγ stimulation in LLC cells. This process may be mediated by the regulatory effects of lycopene on AKT signaling. However, no change in PD-L1 was observed in tumors, suggesting that lycopene could disrupt the induction of PD-L1 by T cell-secreted IFNγ.

## Conclusion

In conclusion, this is the first study discovering the role of lycopene in anti-PD-1 therapy. We have found that lycopene is a promising natural chemical that could synergistically function with anti-PD-1 to prevent tumorigenesis. Its immunomodulatory role assists anti-PD-1 to elevate the levels of IL-1 and IFNγ but reduce the levels of IL-4 and IL-10 in the spleen. In addition to its effects on the CD4+/CD8+ ratio, lycopene also increases the number of IFNγ expressing CD8+ T cells in tumor tissues. Upon IFNγ stimulation, lycopene represses PD-L1 expression via AKT signaling. These results demonstrate that lycopene could be applied to synergistically improve the efficiency of anti-PD-1 therapy.

## Additional file

**Additional file 1: Figure S1.** The photos for all tumors treated by the combined therapy of lycopene and anti-PD-1. **Figure S2.** After the treatment of lycopene and/or PD-1 therapy, the methylation status of IRF3/8 promoter regions were assessed by bisulfite sequencing analysis, (●, methylated cytosine of CpG; ○, unmethylated cytosine of CpG). **Figure S3.** Lycopene and PI3K-AKT inhibitor inhibited AKT signaling to repress expression of PD-L1 in LLC cells.
