# Supplementary material for: Lycopene improves the efficiency of anti-PD-1 therapy via activating IFN signaling of lung cancer cells
Source: Cancer Cell Int. 2019 Mar 21;19:68. doi: 10.1186/s12935-019-0789-y (PMC6429703; doi:10.1186/s12935-019-0789-y)
Supplement: Supplementary file 1 — Additional file 1: Figure S1. The photos for all tumors treated by the combined therapy of lycopene and anti-PD-1. Figure S2. After the treatment of lycopene and/or PD-1 therapy, the methylation status of IRF3/8 promoter regions were assessed by bisulfite sequencing analysis, (●, methylated cytosine of CpG; ○, unmethylated cytosine of CpG). Figure S3. Lycopene and PI3K-AKT inhibitor inhibited AKT signaling to repress expression of PD-L1 in LLC cells. [file 12935_2019_789_MOESM1_ESM.docx]

**Additional file 1**


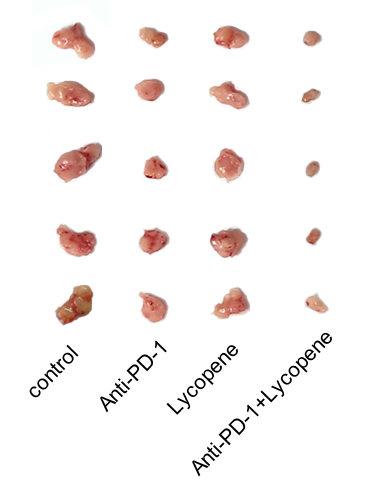


Figure S1: The photos for all tumors treated by the combined therapy of lycopene and anti-PD-1.

**
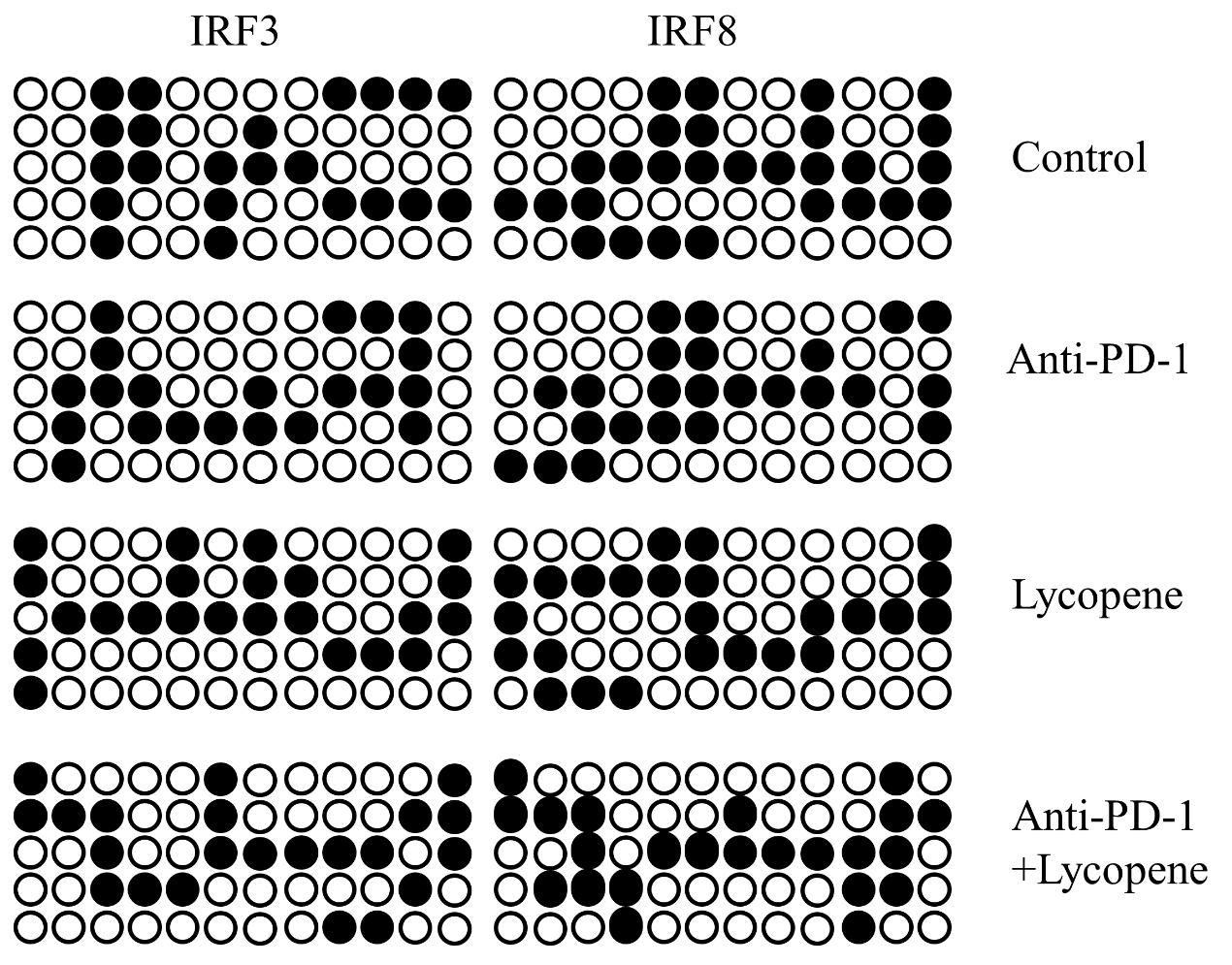
**

Figure S2: After the treatment of lycopene and/or PD-1 therapy, the methylation status of IRF3/8 promoter regions were assessed by bisulfite sequencing analysis, (●, methylated cytosine of CpG; ○, unmethylated cytosine of CpG).

**

**

Figure S3: Lycopene and PI3K-AKT inhibitor inhibited AKT signaling to repress expression of PD-L1 in LLC cells.
